# Channelopathy Genes in Pulmonary Arterial Hypertension

**DOI:** 10.3390/biom12020265

**Published:** 2022-02-07

**Authors:** Carrie L. Welch, Wendy K. Chung

**Affiliations:** 1 Department of Pediatrics, Columbia University Irving Medical Center, New York, NY 10032, USA; cbw13@columbia.edu; 2 Department of Pediatrics, Herbert Irving Comprehensive Cancer Center, Columbia University Irving Medical Center, New York, NY 10032, USA; 3 Department of Medicine, Herbert Irving Comprehensive Cancer Center, Columbia University Irving Medical Center, New York, NY 10032, USA

**Keywords:** channelopathy, genetics, lung disease, pulmonary arterial hypertension

## Abstract

Pulmonary arterial hypertension (PAH) is a rare, progressive vasculopathy with significant cardiopulmonary morbidity and mortality. The underlying pathogenetic mechanisms are heterogeneous and current therapies aim to decrease pulmonary vascular resistance but no curative treatments are available. Causal genetic variants can be identified in ~13% of adults and 43% of children with PAH. Knowledge of genetic diagnoses can inform clinical management of PAH, including multimodal medical treatment, surgical intervention and transplantation decisions, and screening for associated conditions, as well as risk stratification for family members. Roles for rare variants in three channelopathy genes—*ABCC8*, *ATP13A3*, and *KCNK3*—have been validated in multiple PAH cohorts, and in aggregate explain ~2.7% of PAH cases. Complete or partial loss of function has been demonstrated for PAH-associated variants in *ABCC8* and *KCNK3*. Channels can be excellent targets for drugs, and knowledge of mechanisms for channel mutations may provide an opportunity for the development of PAH biomarkers and novel therapeutics for patients with hereditary PAH but also potentially more broadly for all patients with PAH.

## 1. Introduction

Pulmonary arterial hypertension (PAH) is a rare, progressive and often lethal disease characterized by distinctive changes in pulmonary arteries that lead to increased pulmonary vascular resistance [1,2,3]. The estimated prevalence of PAH is 4.8–8.1 cases/million for pediatric-onset [4] and 5.6–25 cases/million for adult-onset disease [5]. Dysregulated vascular, inflammatory, and immune cells contribute to increased vascular resistance and proliferative remodeling of pulmonary arteries and arterioles [3]. These pathological changes increase the load on the right ventricle of the heart, leading to right ventricular hypertrophy initially and, later, right heart failure with high mortality. PAH can occur in families or sporadically. PAH can occur idiopathically or associated with other diseases such as congenital heart disease, autoimmune connective tissue diseases, portopulmonary disease or other rare diseases, or associated with exposure to certain medications/toxins. PAH can present at any age, with more severe disease associated with early age of onset [6]. There is a ~1:3 female to male ratio among young to middle-aged adults diagnosed with disease, suggesting a role for sex hormones in disease etiology. However, the disease incidence is similar for females and males among prepubertal children [6] or older adults (>65 years) [7], suggesting less influence of hormones in childhood and elderly cases.

PAH may be caused by genetic, epigenetic, and environmental factors, as well as gene x environment interactions, wherein genetic contributions to disease risk are modified by environmental exposures. Early genetic studies of heritable PAH (HPAH) in families identified bone morphogenetic protein receptor type 2 (*BMPR2*) as a major causal gene. Genetic data from patient registries and large PAH cohorts indicate that ~70% of familial cases are caused by rare deleterious *BMPR2* variants. However, familial cases represent a small fraction of all PAH cases. Variants in *BMPR2* and other genes in the BMPR2 signaling pathway (*ACVRL1*, *ENG*, *SMAD9*, and *GDF2*) are causal in ~12–20% of sporadic, idiopathic PAH (IPAH) cases and rarely in PAH associated with other diseases (APAH) or medication/toxins [6]. More recently, exome and genome sequencing studies have identified novel PAH risk genes outside of the BMPR2 pathway, including three channel genes: ATP-binding cassette subfamily member 8 (*ABCC8*), ATPase 13A3 (*ATP13A3*), and potassium two-pore domain channel (*KCNK3*). A fourth channel gene, aquaporin 1 (*AQP1*), was reported in one study [8] but has not yet been replicated. Identification of a genetic cause of PAH in individual cases can have implications for clinical management including treatment (mono- vs. multimodal therapy), surgical intervention and transplantation decisions, and screening for associated conditions. At least 13% of adult-onset and 43% of child-onset PAH cases can be explained by genetic causes [9].

This review will focus on the three confirmed channel genes, the genetic variants associated with PAH, and therapeutic implications. Variants included in this review are all ultra-rare (allele frequency < 0.0001, gnomADv2.1.1) and likely gene disrupting (frameshift, stopgain, splicing) or missense with deleterious predictions (CADD ≥ 20). The reference population allele frequency used for each variant is matched to the genetic ancestry of the discovery cohort. Variant residue numbering is based on transcripts NM_001351295.1 (*ABCC8*), NM_0024524.3 (*ATP13A3*), and NM_002246.2 (*KCNK3*). An overview of the genes, frequencies of unrelated cases identified with rare deleterious variants, associated PAH phenotypes and variant types, and observed modes of inheritance is provided in Table 1.

## 2. *ABCC8* (OMIM *600509)

Outside of the TGFβ/BMP pathway, channelopathy gene, *ABCC8*, is one of the most common causes of PAH, accounting for ~1% of cases. *ABCC8* is a member of the ATP-binding cassette (ABC) transporter gene superfamily and encodes the sulfonylurea receptor 1 (SUR1) protein, an ATP-sensitive potassium channel regulatory subunit. Potassium channels play important roles in maintaining cellular resting membrane potential and intracellular calcium concentrations. *ABCC8* is highly expressed in pancreatic islet cells where it functions to release insulin, and recessive mutations cause congenital hyperinsulinemia and neonatal diabetes mellitus [23,24]. *ABCC8* was first identified as a PAH risk gene by exome sequence analysis of a cohort of 99 pediatric- and 134 adult-onset PAH cases [10]. A rare, predicted deleterious de novo missense variant was identified in a 10-year-old patient with IPAH. De novo variants are infrequent and, when deleterious, can be associated with high mortality at an early age and, therefore, are not transmitted to the next generation. Thus, the entire PAH cohort was screened for genetic variants in *ABCC8*, and rare or novel missense variants were identified in seven unrelated patients with IPAH, HPAH or APAH-congenital heart disease (APAH-CHD). Consistent with autosomal dominant inheritance of PAH, all individuals were heterozygous for the rare *ABCC8* variants. The *ABCC8* c.718G>A;p.240A>T variant segregated with PAH in one family. A replication study in a United Kingdom (UK) PAH cohort identified four additional variants, three missense and one splice variant. One *ABCC8* variant heterozygote also had a rare *TBX4* frameshift mutation; and none of the other carriers had mutations in any known risk genes. In the combined US/UK, approximately half of the patients had adult-onset and half pediatric-onset disease. Statistical enrichment analyses of *ABCC8* variants in European PAH cases compared to two independent European population control groups (*n* > 33,000 individuals each), indicated a 3-fold enrichment rate of *ABCC8* variants among PAH cases with no significant difference in the frequency of predicted benign synonymous variants between cases and controls.

Independent studies carried out in large PAH cohorts from the US and Spain and two smaller cohorts have validated *ABCC8*. The US-based National Biological Sample and Data Repository for PAH (aka PAH Biobank) includes 2572 PAH cases with exome sequence data [6]. Rare or novel, predicted deleterious *ABCC8* missense variants were identified in twenty-eight cases. There were twenty-five unique variants, with three recurrent variants (Met400Val, Arg670Cys, Gln830Lys) each identified in two unrelated cases. Additionally, novel missense variant, c.2439T>A;p.Ser813Arg, affects the same amino acid residue as c.2437G>A;p.Ser813Arg reported in Bohnen et al. [10]. The *ABCC8* variants occurred at the same frequency in IPAH and APAH (CHD, connective tissue diseases, and one case with HIV). Ten percent (*n* = 3) of the cases were diagnosed as children, similar in frequency to the overall composition of the cohort (91% adult, 9% children). Panel gene sequencing of 21 PAH genes in 624 PAH cases from the National Spanish PAH Registry identified five missense, one splice, and one frameshift variant were identified in *ABCC8* [11,25]. The splice variant and one missense variant predicted to affect splicing were demonstrated to cause exon skipping in a minigene assay [25]. The splice variants were identified in patients with APAH (CREST, CHD); the frameshift variant carrier had IPAH, and the missense variant heterozygotes had IPAH (*n* = 4) or APAH-CHD (*n* = 1). In a small cohort of pediatric PAH cases (*n* = 18), Gelinas and colleagues identified a novel *ABCC8* missense variant associated with IPAH in an analysis of 26 PAH genes [12]. Finally, in a Chinese cohort of persistent pulmonary hypertension of the newborn (*n* = 74), Liu and colleagues identified a novel stopgain variant, c.2331G>A;p.W777X, with exome sequencing in a newborn male with hypoglycemia [13]. Overall, *ABCC8* variants explain ~1.4% of PAH cases (Table 1), including primary PAH and PAH associated with other diseases.

**Figure 1:**
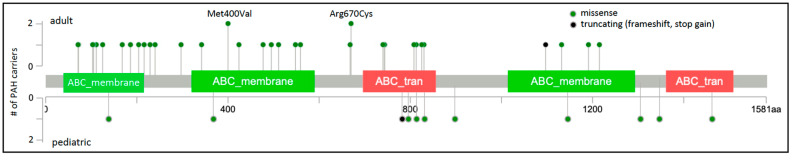
*ABCC8* two-dimensional protein schematic. Variants identified in adult-onset PAH cases are shown above the protein schematic; variants identified in children are shown below. Variant type is color-coded. The number of PAH carriers identified with a particular variant is shown along the *y*-axis. Note that splice variants are not included and variant density may impede visualization of closely-located variants. Conserved protein domains are indicated by colored rectangles. ABC_membrane, ABC transporter transmembrane region; ABC_tran, ABC transporter. Map generated using MutationMapper at cBioPortal.org, accessed on 2 January 2022.

Of the 45 rare, predicted deleterious *ABCC8* variants identified in 49 unrelated PAH cases, 41 are missense variants. The locations of the missense variants, one frameshift, and one stopgain variant are shown in Figure 1. Variants identified in adult- and pediatric-onset PAH are depicted above and below the protein schematic, respectively. The vast majority of the missense variants (32/41) reside in conserved ABC protein transmembrane or nucleotide-binding domains. Three additional variants, c.647G>A;p.Arg216His, c.686C>T;p.Thr229Ile and c.718G>A;p.Ala240Thr, reside in a cytoplasmic loop containing a Lasso motif near the ATP site which may regulate channel activity [26]. Eight of the missense variants have been tested via patch-clamp electrophysiology and rubidium efflux studies of mutant COS cells, and all mutant cells exhibited significantly decreased channel activity [10]. Diazoxide is a SUR1 activator used to treat congenital hyperinsulinemia. Addition of exogenous diazoxide to the *ABCC8* mutant cells at least partially normalized currents and rubidium efflux [10]. The single-frameshift and stopgain variants are predicted to result in nonsense-mediated decay. Some of the PAH-associated *ABCC8* variants have been reported in patients with autosomal recessive juvenile hyperinsulinemia or neonatal diabetes mellitus but none of the heterozygous PAH cases, aside from the case with persistent pulmonary hypertension of the newborn, exhibited derangements in glucose metabolism. Diabetes is a common metabolic comorbidity associated with adult-onset PAH [27]; whether heterozygous variants in *ABCC8* contribute to the co-occurrence of diabetes and adult-onset PAH remains an open question.

ABCC8 is normally expressed in lung tissue, and immunohistochemical studies show that ABCC8 (SUR1) protein is expressed in pulmonary arterial endothelial cells (PAECs) [10] and pulmonary arterial smooth muscle cells (PASMCs) [28]. Ion channel activity in PASMCs is a key determinant of vasoreactivity and cell proliferation, affecting pulmonary vascular tone and remodeling, two important pathogenic mechanisms in PAH. Hypoxia [29] and elevated shear stress [30] induce SUR1 upregulation in rodents. We have shown that *ABCC8* gene expression is increased in PAH patients with *BMPR2* pathogenic variants compared to healthy controls [10]. While the genetic evidence for autosomal dominant, primarily missense, *ABCC8* variants in PAH is well established and replicated in multiple large studies, more experimental evidence is needed to elucidate the pathogenetic mechanism.

## 3. *ATP13A3* (OMIM *610232)

*ATP13A3* encodes a transmembrane cation transporter which was recently shown to transport polyamines [31]. Polyamines are small metabolites required for normal cell growth and proliferation, and elevated plasma concentrations have been reported in multiple cancers and, more recently, PAH [32,33]. Genetic variants in *ATP13A3* were first reported in a genome sequencing study of the UK NIHR Bioresource-Rare Diseases Study of 1083 PAH cases [8]. After excluding cases with variants in seven established PAH causal genes, a higher frequency of protein-truncating variants was observed in cases compared to controls. A trend was also observed for protein-truncating plus missense variants but did not reach genome-wide significance. Ten unrelated IPAH cases with unique rare, predicted deleterious variants were identified: three frameshift, two stopgain, one splice variant, and four missense variants. Independent validation of *ATP13A3* in PAH was demonstrated in several studies worldwide. Four additional missense variants were identified in a Chinese cohort of 331 unrelated IPAH cases [14]. In the European pediatric PAH cohort cited above, one novel missense variant was identified in an APAH-CHD case [12]. Interferon beta is recognized as a cause of drug-induced PAH, often reversible with cessation of treatment [34]. In a study of two multiple sclerosis patients treated with IFNβ-1a and exhibiting reversible PAH [15], Lerche and colleagues identified a novel missense variant, c.1540C>T;p.Gln514X, in one of the patients. In the PAH Biobank, seven unique variants (three frameshift, one stopgain, and three missense) were identified in seven unrelated cases (five HPAH/IPAH, one APAH-CTD, and one APAH-CHD) [6]. In each of these studies, the cases were adult-onset disease, with the exception of one child, and all were heterozygous.

We reported a case series of three families with pediatric-onset severe PAH and early mortality [16]. In total, there were five children diagnosed with PAH under the age of three years, largely refractory to medical treatment, and four died in early childhood. The surviving child underwent a Pott’s shunt to decompress the right ventricle [35]. Exome/genome sequence identified biallelic *ATP13A3* variants in each of the affected children. The unaffected parents were heterozygous for one of the variants, and unaffected siblings were either heterozygous for a variant or homozygous for the reference allele. The variants included two frameshift, one stopgain, and two missense variants. Variants occurring at ATP13A3 amino acid 850 have been reported now in three cases, including three occurrences of frameshift variant c.2549dup; p.Met850Ilefs*13 (a monoallelic adult case [6] and a biallelic childhood case [16]). Further, c.2228G>T;p.Arg743Cys, identified in a biallelic child-onset case, impacts the same amino acid residue as reported for an adult-onset case (c.2227C>T;p.Arg743Cys) [6]. These data are consistent with semi-dominant inheritance for the *ATP13A3* gene. Biallelic inheritance indicates a dose-dependent effect and may have implications for prognosis and aggressive treatment strategies in child-onset PAH.

**Figure 2:**
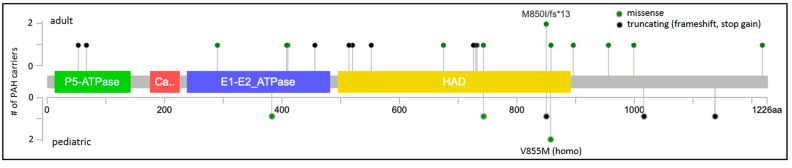
*ATP13A3* two-dimensional protein schematic. Variants identified in adult-onset PAH cases are shown above the protein schematic; variants identified in children are shown below. Variant type is color-coded. The number of PAH carriers identified with a particular variant is shown along the *y*-axis. Conserved protein domains are indicated by colored rectangles. E1-E2_ATPase, cation transporter ATPase; HAD, haloacid dehalogenase-like hydrolase. Map generated using MutationMapper at cBioPortal.org, accessed on 2 January 2022.

The protein locations of the 23 unique *ATP13A3* variants are shown in Figure 2. *ATP13A3* is highly constrained for loss-of-function variants (pLoF = 1) [36], with heterozygous alleles occurring very rarely and no homozygous occurrences reported in gnomAD. Most of the missense variants (7/10) occur in conserved protein domains. A role for ATP13A3 in polyamine transport has only recently been reported [31], and is currently under investigation. *ATP13A3* is widely expressed in the developing embryo and adult tissues [37], including PASMCs [8]. Hypoxia stimulates accumulation of spermine leading to increased PASMC proliferation in model systems [33]. We hypothesize that *ATP13A3* variants predicted to alter transporter function disturb polyamine homeostasis.

The pathogenetic mechanism of *ATP13A3* variants in PAH is under investigation. Both missense and protein truncating variants have been reported in PAH patients. Most of the protein truncating variants are predicted to undergo nonsense-mediated decay indicating haploinsufficiency as the likely mechanism. For the missense variants, it is unclear whether the mechanism is loss or gain of function. In vitro screening of the variants is required to determine whether the variants affect membrane trafficking, polyamine uptake, polyamine release, or polyamine metabolism.

## 4. *KCNK3* (OMIM *603220)

The *KCNK3* gene encodes a two-pore domain potassium channel, also known as TASK1. *KCNK3* was first identified as a PAH causal gene by rare variant analysis of exome sequencing data in a family with five affected members [17]. A c.608G>A;p.Glu203Asp missense variant was identified in four affected and one unaffected family members. An additional unaffected individual did not carry the family variant. To identify additional *KCNK3* variants, exome sequencing data from 92 familial and 230 IPAH cases were screened. Five additional heterozygous missense variants were identified, confirming an autosomal dominant mode of inheritance with incomplete penetrance. None of these *KCNK3* variant heterozygotes had variants in other known PAH risk genes. Patch clamp studies in COS-7 cells expressing the mutant proteins indicated loss of potassium channel function that could be partially rescued by treatment with a phospholipase inhibitor [17]. Similarly decreased channel current resulted from expression of mutant channels alone or co-expression of both mutant and wild-type channels.

**Figure 3:**
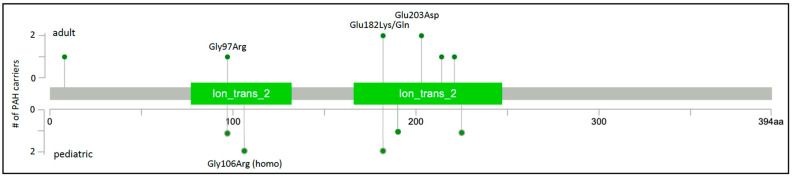
*KCNK3* two-dimensional protein schematic. Variants identified in adult-onset PAH cases are shown above the protein schematic; variants identified in children are shown below. All variants are missense. The number of PAH carriers identified with a particular variant is shown along the *y*-axis. Conserved protein domains are indicated by colored rectangles. Ion_trans_2, ion channel. Map generated using MutationMapper at cBioPortal.org, accessed on 2 January 2022.

Rare missense variants in *KCNK3* were subsequently reported in at least six additional studies of HPAH and IPAH. Two novel variants were identified in a Spanish cohort of 136 unrelated cases [18]. One of the cases, a severe form of PAH diagnosed at 2 months of age, was from a consanguineous family and was homozygous for the c.316G>C;p.Gly106Arg variant His-affected mother was heterozygous for the variant and diagnosed at 19 years of age. Functional analysis of the variants showed similar membrane localization and loss of channel function that could not be rescued by pharmacologic activators of TASK-1 channels [38]. An exome/genome sequencing analysis of nine Japanese families with PAH identified one rare missense variant in one family [19], c.608G>A;p.Glu203Asp, a recurrence of the variant first observed in a family from Ma et al. [17]. Screening of 82 Chinese pediatric PAH cases [20] revealed another recurrent *KCNK3* variant, c.289G>A;p.Gly97Arg, also reported by Ma et al. [17]. Exome sequence analysis of 412 largely European pediatric and adult cases with HPAH or IPAH identified one case with a novel variant, c.675C>A;p.Phe225Ile, and one case with recurrent variant, c.544G>A;p.Glu182Lys [21]. A single occurrence of the c.544G>A;p.Glu182Lys variant was also identified in the PAH Biobank [6]. Finally, a different coding variant affecting the same amino acid residue, c. 544G>A;p.Glu182Gln, was identified in a large rare disease cohort from the UK [22]. A total of nine variants in fourteen cases, with four recurrent variants identified in at least two cases each, were identified in these seven studies. Together, the variants, all missense, explain ~0.3% of HPAH/IPAH (Table 1). The locations of the nine PAH-associated *KCNK3* variants are depicted in Figure 3; all but one variant maps to one of two conserved ion transport domains. The high frequency of recurrence of PAH-associated *KCNK3* variants is striking, underscoring the importance of these residues in pulmonary vascular dysfunction. While a semi-dominant mode of inheritance is indicated in the consanguineous family with very early-onset severe PAH, segregation data in all other families indicate an autosomal dominant mode.

*KCNK3* is widely expressed, including in PASMCs, where it plays a role in regulating resting membrane potential and pulmonary vascular tone. Antigny and colleagues [39] demonstrated decreased lung and pulmonary artery expression of *KCNK3* in freshly isolated PASMCs from IPAH patients and HPAH patients with *BMPR2* variants compared to unaffected controls; and decreased channel conductance in IPAH vs. control PASMCs. Moreover, HPAH cases with *BMPR2* variants also had decreased *KCNK3* lung expression. Murine KCNK3 does not form a functional ion channel in PASMCs, being replaced by KCNK6 [40,41]. To gain a better understanding of the role of *KCNK3* in PAH, transgenic rats expressing a mutant TASK-1 channel were developed using CRISPR [42]. The rats have a 94 bp deletion in *Kcnk3* exon 1, leading to expression of a dysfunctional protein rather than nonsense-mediated decay. Decreased channel conductance in PASMCs leads to age-related increase in right ventricular systolic pressure, sensitization to monocrotaline-induced PH, and muscularization of distal pulmonary arterioles in this transgenic rat model. The rats are resistant to pulmonary artery relaxation mediated by the vasodilator, sildenafil [42]. The loss-of-function mutant rat model also develops more severe PH in response to ascending aortic constriction [43], suggesting a broader role for KCNK3 in PH Group 2/left heart disease as well as PH Group 1/PAH. The model may be valuable for pharmacological testing of potential treatments for PH.

## 5. Other Channel Genes Associated with PAH

*KCNA5* (OMIM *176267) encodes Kv1.5, a voltage-gated potassium channel activated by membrane depolarization and regulating voltage-gated calcium channel activity [44,45]. Kv1.5 is expressed in smooth muscle cells of several tissues including human and rodent PASMCs. PAH patients with *BMPR2* mutations have decreased *KCNA5* lung expression and exogenous treatment of isolated human PASMCs with BMP2 normalizes gene expression [46]. Common single-nucleotide polymorphisms in coding and non-coding regions of *KCNA5* have been reported with increased frequency in PAH cases compared to controls [47,48,49], suggesting that *KCNA5* variants could modulate PAH onset and severity. However, the *KCNA5* association with PAH has been refuted in other reports [50], including a recent literature-based meta-analysis [51], and was not observed in a large European meta-analysis [52].

*ABCC9* (OMIM *601439) encodes SUR2, sufonylurea receptor 2, another regulatory subunit of ATP-sensitive potassium channels. *SUR2* is expressed in vascular smooth muscle cells, primarily in the heart [53]. Rare gain-of-function *ABCC9* variants cause Cantu Syndrome characterized by hypertrichosis, osteochondroplasia, cardiac defects including cardiomegaly, and other abnormalities. Some Cantu patients with *ABCC9* variants have also been diagnosed with PH [54,55,56]. However, *ABCC9* variants have not been reported in PAH cohorts.

*TRPC6* (OMIM *603652) encodes transient receptor potential cation channel, subfamily C, member 6. TRP channels are important regulators of intracellular cation homeostasis with TRPC6 being especially important in regulating Ca^++^ levels. *TRPC6* is ubiquitously expressed in the vasculature and with prominent expression in PASMCs of distal arterioles. Activation of TRPC6 channel activity in PASMCs gives rise to elevated intracellular Ca^++^ concentrations and is associated with hypoxia-induced vasoconstriction which is abolished with TRPC6 deficiency [57]. Rare inherited variants in *TRPC6* cause focal, segmental glomerulosclerosis [58] but have not been reported for PAH. A promoter-activating common single-nucleotide polymorphism in *TRPC6*, -254C>G, was associated with IPAH in a study of 268 IPAH cases [59] but was not identified in a recent meta-analysis of 2085 PAH cases [52].

## 6. Therapeutic Implications

Current PAH therapies target vasoactive pathways to treat symptoms and delay disease progression, but none are curative. There is a crucial need for new therapies aimed at inhibiting or reversing pulmonary vascular remodeling to stop disease progression and increase survival. Ion and small-metabolite channels play important roles in cellular homeostasis, sensing and reacting to changes in the local microenvironment. In the pulmonary vasculature, changes in channel function caused by genetic variants, pharmaceutical interventions, or local environmental factors, can alter vascular tone, cell proliferation, and cell metabolism. Potassium ion channel dysfunction has been documented in PASMCs from PAH patients with gene variants in *ABCC8* and *KCNK3*. Altered *ABCC8* and *KCNK3* expression has also been reported in IPAH cases without known channel gene variants, suggesting that therapeutic modulation of these genes or channels may be broadly effective for PAH. *TRPC6* expression is elevated in IPAH independent of genetic mutations and selective inhibitors are in development that require testing in animal models [57]. A large metabolomic study identified increased levels of circulating polyamine metabolites as a potential biomarker for PAH, with increased survival for patients with improved metabolite profiles over time [32]. These data suggest that regulation of ATP13A3 channel activity/expression may also be beneficial to PAH patients with or without *ATP13A3* variants; however, more research is needed regarding the functional role of ATP13A3 in PAH.

Plasma membrane channels can be targeted pharmaceutically with selective activators or inhibitors of channel function. However, such treatment can lead to unwanted side effects. For example, the development of PH in hyperinsulinemic hypoglycemia patients treated with the SUR1 activator, diazoxide [60,61,62,63]. Further, the association of PAH with *KCNK3* and *ABCC8* variants is mediated by missense loss-of-function variants. Severe loss of function may not be ameliorated by channel activators [38]. Thus, molecular genetic therapy approaches may be considered in the future, including gene addition or upregulation of the normal allele in PASMCs. Successful gene transfer of *KCNA5* in a rat model restored potassium channel current, normalized hypoxic pulmonary vasoconstriction, and ameliorated PH [64]. Alternatively, knockdown of a mutated allele by targeted siRNA may be necessary for alleles with a dominant negative effect. The application of such genetic therapies in humans awaits longer-term safety and efficacy studies from other diseases. The lungs and pulmonary vasculature are accessible tissues when safety and durability have been addressed.

## 7. Summary

Our understanding of the genetic architecture of PAH continues to evolve, with new candidate causal genes, new genetic variants, and additional modes of inheritance being identified. Roles of genetic variants in three channelopathy genes—*ABCC8*, *ATP13A3*, and *KCNK3*—have been validated in multiple independent studies, and explain ~2.7% of PAH cases. Loss-of-function missense variants in *ABCC8* are the most common (1.4% of cases), likely causal in both H/IPAH and APAH cases. A gene dosage effect has been demonstrated for *ATP13A3*, with biallelic variants causal for very early-onset severe PAH with high mortality. The molecular mechanism for *ATP13A3* variants in PAH (i.e., loss of function, gain of function, and haploinsufficiency) is not yet known. Loss-of-function variants in *KCNK3* (missense only) are rare causes of PAH. The role of these genes in the broader spectrum of PH requires further study. Knowledge of causal mechanisms in channelopathies may provide an opportunity for the development of novel therapeutics based on inhibition or augmentation of channel function.

## Figures and Tables

**Table 1:** PAH causal channelopathy genes and associated variant allele frequencies/characteristics across multiple studies.

Gene	Gene Name	Number of Cases (%)	PAH Subclass ^1^	Variant Type ^2^	Mode of Inheritance ^3^
*ABCC8*	ATP-binding cassette subfamily C member 8	49/3521 (1.4%) [6,10,11,12,13]	**H/I/APAH** APAH-CTD APAH-CHD APAH-HIV PPHN	**missense**, LGD	AD
*ATP13A3*	ATPase 13A3	27/4012 (0.7%) [6,8,12,14,15,16]	**H/IPAH** APAH-CTD APAH-CHD APAH-MS/IFNβ-1a	LGD, missense	Semi-dominant
*KCNK3*	Potassium two-pore domain channel subfamily K member 3	14/4682 (0.3%) [6,17,18,19,20,21,22]	H/IPAH	missense	AD

^1^ H/IPAH, hereditary or idiopathic pulmonary arterial hypertension; APAH-CTD, PAH associated with connective tissue diseases; APAH-CHD, PAH associated with congenital heart disease; PAH associated with HIV; PPHN, persistent pulmonary hypertension of the newborn; APAH-MS/IFNβ-1a, PAH associated with interferon beta 1a treatment in multiple sclerosis; APAH-porto, PAH associated with portopulmonary disease. Bold typeface indicates primary PAH subclass. ^2^ Variants filtered by gnomAD allele frequency < 0.0001 and variant type likely gene disrupting (LGD, stopgain, frameshift, splicing) or damaging missense defined by CADD ≥ 20. Bold typeface indicates primary variant type. ^3^ MOI, mode of inheritance; AD, autosomal dominant.

## Data Availability

Not applicable.
